# Advancing tele‐physiology: A chest patch solution for continuous, non‐invasive remote monitoring in a hypoxic environment

**DOI:** 10.1113/EP093718

**Published:** 2026-05-30

**Authors:** Danilo Bondi, Ferdinando Calcagno, Elisa Fioraso, Camillo Di Giulio, Federico Schena, Anna Pedrinolla

**Affiliations:** ^1^ Department of Neurosciences, Imaging and Clinical Sciences University ‘G. d'Annunzio’ Chieti ‐ Pescara Chieti Italy; ^2^ Department of Engineering for Innovation Medicine University of Verona Verona Italy; ^3^ Department of Neurosciences, Biomedicine and Movement Sciences University of Verona Verona Italy; ^4^ Sport, Mountain and Health Research Centre (CeRiSM) University of Verona and University of Trento Rovereto Italy; ^5^ Department of Science University “G. d'Annunzio” Chieti ‐ Pescara Chieti Italy; ^6^ Department of Cellular, Computational and Integrative Biology (CIBIO) University of Trento Trento Italy

**Keywords:** chest patch, high‐altitude, normobaric hypoxia, remote monitoring, skin‐mounted sensors, telemedicine, wearable sensors

## Abstract

Skin‐mounted sensors are thin, flexible and lightweight electronic devices that enable monitoring of multiple physiological parameters. The aim of this study was to provide pilot evidence for possible utilization of a sensorized patch for capturing the bodily response to hypoxia. The subsequent phases of the study comprised a case report and a case series, with participants undergoing physical tests in both normobaric normoxia and hypoxia. The data obtained from VitalPatch® were compared with data from the metabolic cart COSMED Quark CPET. In the case report, agreement was substantial for heart rate (HR) and poor for respiratory rate (RR) in an incremental normoxic test, and almost perfect for HR and moderate for RR in a time‐to‐exhaustion hypoxic test; in the case series, agreement was substantial for HR and poor for RR. The trend of values generally was almost superimposable and mean bias was overall acceptable. ECG alarms were considered mostly false or highly uncertain, possibly due to noise and artifacts. The quality of the recording was unaffected by the presence of hypoxia. VitalPatch® is best used at rest and in minimal movement, offering reliable HR and RR monitoring.

## Abstract (highlights)

- **What is the central question of this study?**
  Can a sensorized patch accurately capture the bodily response to hypoxia during physical exercise?
- **What is the main finding and its importance?**
  The VitalPatch^®^ sensor is suitable for field applications under hypoxic conditions, but the presence of motion artifacts still limits its use during hypoxic exercise.

## 1 INTRODUCTION

High‐altitude expeditions are a large growth sector, whether for tourism, business or sports purposes. To enable high‐risk patients to fully enjoy the experience while mitigating risks, and generally to improve the safety of those who expose themselves to such hypoxic environments, the use of reliable sensors for remote monitoring could be of enormous value. By using wearable/portable sensors and Internet of Things (IOT) technology, it is currently possible to obtain real‐time data of physiological characteristics of expeditioners, their positions and the features of the surrounding environment, thereby enabling continuous monitoring and timely communication, reducing response times and increasing safety in challenging and remote locations (Donavalli et al., 2024; Thendral et al., 2024). Such sensors should ensure duration, portability and ease of handling by the subject, robustness and resistance to harsh conditions, automatic alarms, data handling, and comprehensive reports. The real time data‐processing of vast amounts of data generated by advanced biosensors enables anomaly detection, timely interventions and personalized health insights (Raymond et al., 2024). Physiological datasets of several research topics need expansion into real‐world environments, with cost‐effective devices that can be utilized even with no specialized knowledge (Cobá Juárez Pegueros & Rodríguez‐Arce, 2025).

Due to their non‐invasive nature and the possibility of continuous, real‐time data acquisition and analysis, sensorized patches with embedded biosensors have gained growing attention for remote health monitoring, fitness monitoring and medical research (Mali et al., 2023). These devices, also known as skin‐mounted sensors, are thin, flexible and lightweight electronic devices that adhere directly to the skin and can integrate a variety of biosensing technologies, thereby allowing monitoring of several physiological parameters. However, these biosensors are usually utilized in stationary setting with patients, and performance criteria during body movement or outdoor environments are scarce (Areia et al., 2021). More demanding challenges and limitations of using wearable sensors in unusual or extreme environment create issues of accuracy and reliability of measurement, signal quality, motion artifacts, comfort and usability, integrity and transmission of data, user acceptance, power consumption and battery life (Chen et al., 2023; Liu et al., 2024; Shen et al., 2020).

Given the growing number of individuals travelling to high altitudes, sensorized patches in this extreme environment would be highly useful. Indeed, these biosensors reduce human‐related limitations and challenges of wearable sensors in extreme environments, such as discomfort and direct exposure to the external environment, but technical challenges may limit their use. Indeed, exposure to cold temperatures, hypobaria, UV radiation, low humidity, special clothing, dirt, sweat, interferences and body movements are all likely to affect the devices' functions. The operating conditions of one such device, VitalPatch®, are as follows: temperature range of 0–40°C, storage relative humidity range of 10–95%, operating duration of 120–168 h, altitude < 3000 m, and barometric pressure of 525–765 mmHg.

Therefore, this study aimed to create pilot evidence for using VitalPatch® to capture the bodily response to hypoxia exposure and for adopting this small (120 mm x 41 mm x 9.5 mm) and light (13 g) sensorized patch during subsequent high‐altitude studies.

## 2 METHODS

### 2.1 Study details

#### 2.1.1 Design of the study

This pilot study included two highly stressful physical tests, that is, incremental and time‐to‐exhaustion, in both normobaric normoxia and normobaric hypoxia. We conducted the study in two phases: (1) the first on one participant, before and after a high‐altitude expedition (case report); (2) the second, on four other ad hoc recruited participants who underwent the test sessions on two separate days, interspersed with a day of rest (case series). In all cases, the sensor was kept for at least 2.5 days, from the beginning of the tests in normoxia to the end of the tests in hypoxia; given the availability of participants, it was also kept on the following days until the battery was exhausted.

A physician was present during all exercise tests to ensure participant safety and to provide clinical oversight throughout the protocol. All recorded traces and alarms reported from VitalPatch® (hereinafter referred to as VITALPATCH) were analysed offline by separate physicians with expertise in sports cardiology who were not present during the experimental sessions; the evaluators were blinded to the experimental setting.

#### 2.1.2 Ethical approval

Participants signed their written informed consent. The protocol was approved by the Ethics Committee of the University of Trento (protocol no. 2025–038) and conformed to the 2013 revision of the *Declaration of Helsinki*.

### 2.2 Participants

#### 2.2.1 Case report

A 37‐year‐old male (height: 170 cm; weight: 65 kg; ${\dot{V}}_{O_{2}\max}$: 46.7 mL/min/kg) was included in this case report. At the age of 28, he developed a severe form of meningitis complicated by meningococcal sepsis, septic shock and purpura fulminans, resulting in the amputation of both lower limbs and seven fingers. One year later, he began running with carbon fiber prosthetic blades, and the following year he resumed mountaineering with the aid of specialized prosthetics. He achieved a progressive increase in altitude, reaching the summit of Mount Everest. The subject visited the laboratory twice, completing the four experimental sessions before and after a 15‐day expedition, during which he successfully reached the summit of Mount Denali (6190 m; formerly Mount McKinley, Alaska, USA). The participant wore the body patch before the expedition, during the incremental test in normoxia and in the following days, and after the expedition, during the time‐to‐exhaustion test in hypoxia and in the following days.

#### 2.2.2 Case series

The participants were recruited from among mountain guides or aspiring mountain guides who underwent testing at the Sport, Mountain and Health Research Centre (CeRiSM, Rovereto, Italy) to obtain relevant information about their health and fitness in relation to their work. The sex distribution of this type of worker is notoriously very asymmetrical, with almost all of them being male. Four healthy male subjects were included in the case series (age: 42.0 ± 2.4 years; height: 177.3 ± 5.4 cm; weight: 76.0 ± 9.4 kg; ${\dot{V}}_{O_{2}\max}$: 50.2 ± 8.0 mL/min/kg). All participants were well‐trained and had experience with high‐altitude environments, including various expeditions above 5000 m.

### 2.3 Procedures

#### 2.3.1 Normobaric normoxia and normobaric hypoxia

Both experimental sessions were conducted in an environmental chamber designed to simulate high‐altitude exposure (i.e., normobaric hypoxia). The normoxic condition was established by maintaining ambient air without altering the oxygen concentration (20.9%). The hypoxic condition was set over the reported operating range of VitalPatch® (i.e., <3000 m altitude) and was achieved by reducing the oxygen concentration to 11.3% using an oxygen dilution system based on the vacuum pressure swing adsorption principle (B‐Cat, Tiel, The Netherlands), simulating an altitude of approximately 5000 m above sea level. Temperature was maintained at 22.0 ± 0.5°C using a dedicated air conditioning and refrigeration system (Frigotherm Ferrari SRL, Lana, Italy). Relative humidity was set at 40% in both conditions.

#### 2.3.2 Incremental test

The incremental test was performed on a reclined cycling ergometer (Ergoselect 1200, Ergoline GmbH, Bitz, Germany) and consisted of two phases. The first phase included several 4‐min bouts with 2 min of rest between each bout. Starting at 50 W, the workload was increased by 25 W at each consecutive bout until the subject reached a blood lactate concentration of 4 mmol/L. Once this point was reached, the subject rested for 5 min. Immediately afterward the second phase started, and a ramp test was performed, starting from the workload corresponding to the bout preceding the 4 mmol/L threshold. During the ramp test, the intensity was increased by 25 W every minute until exhaustion.

#### 2.3.3 Time‐to‐exhaustion test

The time‐to‐exhaustion test was performed on the same reclined cycling ergometer used for the incremental test. The test intensity was set at 80% of the ${\dot{V}}_{O_{2}\max}$ determined from the incremental test. After a 3‐min warm‐up at 35–40% of ${\dot{V}}_{O_{2}\max}$, the workload was immediately increased to 80%, and the subject was instructed to maintain a cadence between 80 and 90 rpm until exhaustion. The research staff provided strong verbal encouragement throughout the test. Exhaustion was defined as the point at which the subject was unable to maintain the target cadence despite continuous encouragement.

### 2.4 Instruments

#### 2.4.1 Metabolimeter

Both incremental and time‐to‐exhaustion tests were performed using a metabolic cart (Quark CPET, COSMED, Rome, Italy) integrated with a 12‐lead electrocardiogram (ECG) system. The device measures breath‐by‐breath gas exchange parameters through a mask, including oxygen uptake (${\dot{V}}_{O_{2}}$), carbon dioxide production (${\dot{V}}_{CO_{2}}$), minute ventilation (${\dot{V}}_{E}$) and respiratory exchange ratio (RER), with real‐time data acquisition. Prior to each test, the system was calibrated using reference gases and a 3‐L syringe according to the manufacturer's specifications. ECG monitoring was conducted continuously throughout the test to assess cardiac electrical activity and detect potential arrhythmias or ischaemic changes.

#### 2.4.2 Sensorized patch

The remote monitoring was performed by using a sensorized patch, that is, VitalPatch® (VitalConnect, Inc., San Jose, CA, USA), a wireless, single‐use, battery‐operated biosensor attached on the upper left chest. It allowed recording of electrocardiography (ECG), temperature and activity, thereby computing the R‐R interval, heart rate (HR) variability, respiratory rate (RR), step count, body posture and fall detection. The device allows registration of data for up to 7 days, with the following measurement ranges: ECG from −10 to 10 mV, HR from 30 to 200 bpm, RR from 4 to 42 bpm (Mean Absolute Error, MAE < 1.5 bpm) and body temperature from 32°C to 42°C (MAE < 1°C). An additional adhesive overlay was applied. Data collected were continuously transmitted to a dedicated online platform via a Bluetooth‐paired smartphone. The sensor was developed for the purpose of remote monitoring of patients.

### 2.5 Data analysis

#### 2.5.1 Case report

Before the expedition, data registered during the normoxic incremental tests were checked and aligned by starting time. Although the volunteer reached values of RR and HR around 80 and 180 rpm, respectively, the VITALPATCH device is calibrated to obtain values up to the threshold of 42 breaths per minute, that is, any actual value above 42 bpm is incorrectly interpreted by the system as 42 bpm. Consequently, the final two steps of the incremental test were excluded from the subsequent device comparison, leaving 31 min of recording. Data from both COSMED and VITALPATCH were filtered by using a locally weighted scatterplot smoothing (LOWESS) method, with fine smoothing (20 points in smoothing window), to obtain uniformly 22,644 points (i.e., *f* ≈ 12.17 Hz) despite the diverse sampling frequencies of the raw data between the devices. Once back from the expedition, data registered during the hypoxic time‐to‐exhaustion test were checked and aligned, then values over 42 breaths/min were excluded thereby leaving 5 min and 33 s of recording; data were filtered with fine LOWESS to obtain uniformly 4324 points (i.e., *f* ≈ 12.98 Hz). Data filtering and calculation of mean bias and area under curve (AUC) were carried out with the software GraphPad Prism v. 10.1.1 (GraphPad Software, Boston, MA, USA); Lin's CC calculation (Lin, 1989), along with scale shift, effect size and accuracy, was carried out with the online tool available at https://www.statstodo.com/LinCCC.php. The other correlation measures were obtained by using the software Jamovi v. 2.3.21.0. Before the expedition, from the day between hypoxic and normoxic tests onwards for another 4 days, and after the expedition, from the day of hypoxic test onwards for another 5 days, ECG data from VITALPATCH were analysed through the linked systems *MediBioSense HealthStream* and *ViewECG v.1.1.6*, thus obtaining 24‐h reports of ECG and Heart Rate Variability (HRV) to be downloaded. Atypical episodes from ECG reports were checked.

#### 2.5.2 Case series

The case report was used to identify a feasible approach for further analysis in a case series. To this extent, data registered from COSMED and VITALPATCH were processed with Python, by applying the following steps:

- extraction of data and alignment by time, with an iterative approach supported by visual check;
- imputation of VITALPATCH data with a LOWESS filter (cubic, usually 0.001 fraction);
- applying a third‐order Savitzky–Golay filter with adjustable window (usually 10–15);
- resampling data with *f* = 1 Hz with a linear filter;
- applying a third‐order Savitzky–Golay filter;
- extracting 25 min for the incremental test and 5 min for the TTE test, with an iterative approach supported by visual check and considering the maximum threshold of 42 bpm for RR computed by VITALPATCH system;
- computing AUC, Spearman's correlation coefficient ρ with *P*‐value, Lin's CCC, mean bias and limits of agreement (LoA; ±1.96 × SD);
- graphing pairwise comparison by highlighting the difference area

The full code is provided as Supporting information.

## 3 RESULTS

### 3.1 Case report

**FIGURE 1:**
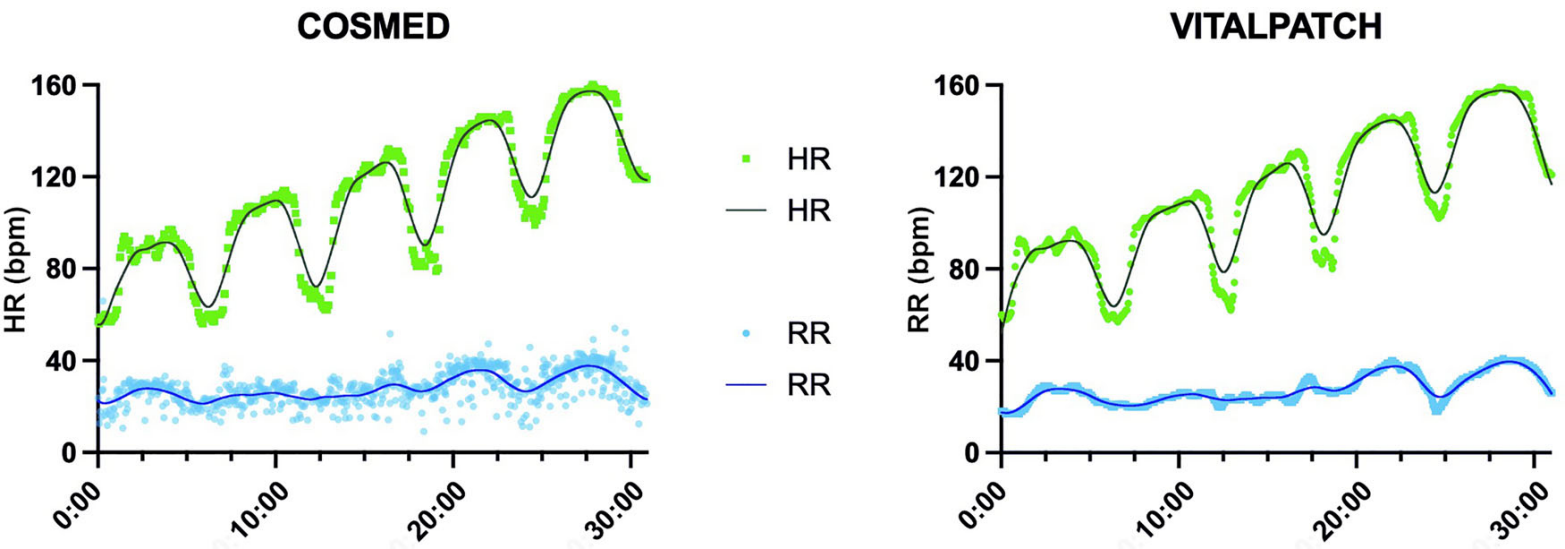
Comparison of data registered with COSMED and VITALPATCH devices during the incremental test in normoxia. Scatter points represent raw data, and the fine smoothing lines were obtained by running a LOWESS method. Data included several 4‐min bouts with 2 min of rest between each bout.

The strength of agreement (McBride, 2005) across methods was considered substantial for HR and poor (although very close to moderate) for RR in the incremental normoxic test; the AUC of curves filtered from VITALPATCH data was 2.40% greater and 0.93% lower for HR and RR, respectively (Figure 1 and Table 1). A previous validation of VITALPATCH data to HR derived from three‐lead ECG and RR determined from capnography measurements (Areia et al., 2021) revealed HR to have a mean bias of 0.31 (95% LoA −3.80 to 4.42) bpm, a precision (Pearson's *r*) of 0.995 during rest, a mean bias of 1.92 (95% LoA −8.75 to 12.59) bpm and a precision of 0.975 during the sit‐to‐stand task, while RR had a mean bias of −1.94 (95% LoA −6.68 to 2.79) bpm, a precision of 0.849 during rest, a mean bias of 1.59 (95% LoA −10.7 to 7.53) bpm and a precision of 0.748 during the sit‐to‐stand task.

**TABLE 1:** Comparison of data registered from COSMED and VITALPATCH devices during the normoxic incremental test, before the expedition.

		HR	RR
Bias	Mean (COS − VIT)	−2.627	−0.260
	95% LoA	−10.58, 5.323	−4.920, 5.439
AUC	COS	20,3516	51,843
	VIT	208,402	51,360
Precision	*r*	0.989	0.894
	ρ	0.989	0.872
	τ	0.920	0.688
Scale shift	ω	0.990	1.276
Effect size	υ	0.098	0.051
Accuracy	χ_a_	0.995	0.970
Lin's CCC	ρ_c_	0.984	0.867
	95% CI	0.983, 0.984	0.864, 0.869

All correlation measures were statistically significant with *P *< 0.001. Abbreviations: AUC, area under curve; CCC, concordance correlation coefficient; CI, confidence interval; COS, COSMED data; HR, heart rate; LoA, limits of agreement; *r*, Pearson's *r*; RR, respiratory rate; ρ, Spearman's rho; VIT, VITALPATCH data; τ, Kendall's tau.

**FIGURE 2:**
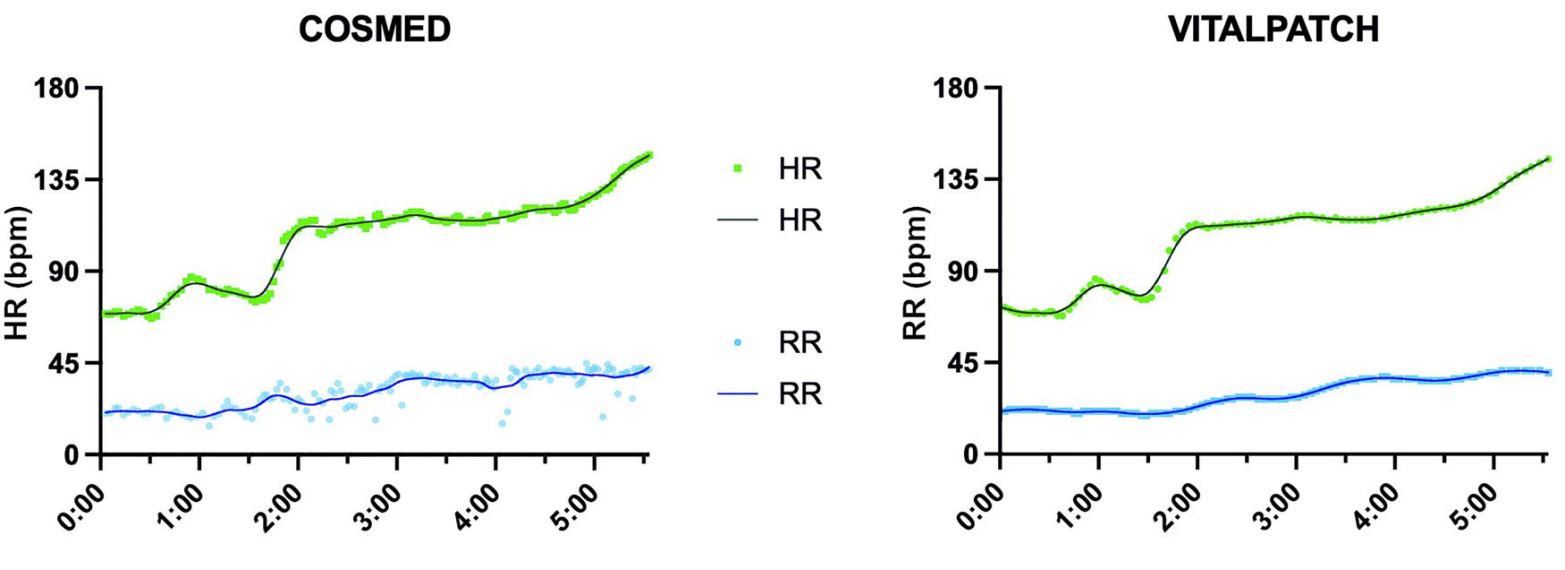
Comparison of data registered with COSMED and VITALPATCH devices during the time‐to‐exhaustion test in hypoxia. Scatter points represent raw data, and the fine smoothing lines were obtained by running a LOWESS method. Data included the warm‐up followed by the workload.

The strength of agreement (McBride, 2005) across methods was considered almost perfect for HR and moderate for RR in the TTE hypoxic test; the AUC of curves filtered from VITALPATCH data was 0.30% greater and 3.78% lower for HR and RR, respectively (Figure 2 and Table 2). A previous validation of VITALPATCH data to HR derived from a three‐lead ECG and RR determined from capnography measurements (Areia et al., 2021) revealed HR to have a mean bias of 0.49 (95% LoA −1.58 to 2.56) bpm and a precision (Pearson's *r*) of approximately 1 during hypoxia exposure with participants gradually desaturating down to 80% $S_{pO_{2}}$, while RR had a mean bias of −0.08 (95% LoA −5.80 to 5.65) bpm and a precision of 0.849.

**TABLE 2:** Comparison of data registered from COSMED and VITALPATCH devices during the hypoxic test, after the expedition.

		HR	RR
Bias	Mean (COS − VIT)	−0.3215	1.151
	95% LoA	−4.767, 4.124	−4.884, 7.186
AUC	COS	35,171	10,132
	VIT	35,278	9749
Precision	*r*	0.994	0.916
	ρ	0.991	0.860
	τ	0.931	0.638
Scale shift	ω	1.005	1.020
Effect size	υ	0.015	0.153
Accuracy	χ_a_	>0.999	0.988
Lin's CCC	ρ_c_	0.994	0.905
	95% CI	0.994, 0.995	0.900, 0.911

All correlation measures were statistically significant with *P *< 0.001. Abbreviations: AUC, area under curve; CCC, concordance correlation coefficient; CI, confidence interval; COS, COSMED data; HR, heart rate; LoA, limits of agreement; *r*, Pearson's *r*; RR, respiratory rate; ρ, Spearman's rho; VIT, VITALPATCH data; τ, Kendall's tau.

From ECG inter‐day comparisons, we noticed that on some days (16 April, 14 June and 15 June) summary data of ECG intervals (Tables 3 and 4) lacked full plausibility. The days on which the volunteer performed comparable activities with data acquisition without too many missing points were the day of the normoxia test and the following day. A comparison of the ECG data and the HRV indices before and after the expedition revealed no clear differences, with heterogeneity of trends between the indices and some data almost overlapping. Furthermore, clear trends in the indices on the days following the tests could not be demonstrated, which was also due to differences in the activities carried out on those days.

**TABLE 3:** ECG‐derived metrics from VITALPATCH data before the expedition.

		16 April	17 April	18 April	19 April	20 April
	Duration	06:14:22	11:19:36	17:24:43	11:19:50	13:33:47
	*n* beats	22,422	36,022	55,419	39,242	43,625
	Mean HR (bpm)	62	55	54	59	55
ECG shape	Mean QRS (ms)	77	95	102	106	109
	Mean QT_c_ (ms)	273	333	352	364	366
	Mean PR (ms)	78	130	156	163	169
HRV metrics	SDNN (ms)	164	263	180	186	250
	RMSSD (ms)	61	60	61	57	73
	pNN50	31%	34%	38%	31%	41%
	LF peak (ms^2^)	2721 (0.066 Hz)	2756 (0.097 Hz)	1556 (0.059 Hz)	2224 (0.056 Hz)	2951 (0.066 Hz)
	HF peak (ms^2^)	770 (0.244 Hz)	357 (0.297 Hz)	841 (0.314 Hz)	812 (0.347 Hz)	746 (0.255 Hz)
	LF/HF	3.57	7.72	1.85	2.74	3.96
	DFAα1	1.13	1.17	0.85	0.91	1.00
	DFAα2	1.05	0.79	1.14	1.01	0.84
Atypical beat type	PAC	5	1	7	0	6
	PVC	0	0	1	0	0
	AB	0	0	0	0	0

Abbreviations: AB, atrial bigeminy; DFA, detrended fluctuation analysis; HR, heart rate; LF, low‐frequency band; HF, high‐frequency band; PAC, premature atrial contractions; pNN50, percentage of successive RR intervals that differ by more than 50 ms; PVC, premature ventricular contractions; RMSSD, root mean square of successive RR interval differences; SDNN, standard deviation of NN intervals.

**TABLE 4:** ECG‐derived metrics from VITALPATCH data after the expedition.

		10 June	11 June	12 June	13 June	14 June	15 June
	Duration	07:42:09	19:45:42	16:54:46	13:16:19	07:19:23	10:17:04
	*n* beats	30,867	63,680	57,156	42,318	25,730	35,411
	Mean HR (bpm)	69	54	57	54	60	58
ECG shape	Mean QRS (ms)	96	100	102	98	42	55
	Mean QT_c_ (ms)	334	353	355	343	146	189
	Mean PR (ms)	125	131	133	112	22	38
HRV metrics	SDNN (ms)	198	190	286	222	164	221
	RMSSD (ms)	43	66	62	66	55	55
	pNN50	17%	41%	34%	39%	30%	28%
	LF peak (ms^2^)	946 (0.033 Hz)	1303 (0.048 Hz)	1947 (0.054 Hz)	2290 (0.063 Hz)	2579 (0.085 Hz)	2702 (0.079 Hz)
	HF peak (ms^2^)	386 (0.288 Hz)	929 (0.284 Hz)	882 (0.307 Hz)	878 (0.375 Hz)	307 (0.349 Hz)	460 (0.280 Hz)
	LF/HF	2.45	1.40	2.21	2.61	8.39	5.87
	DFAα1	1.19	0.64	0.55	1	0.84	1.1
	DFAα2	1.08	1.12	0.91	−1.51	1.12	0.71
Atypical beat type	PAC	4	29	2	2	0	2
	PVC	6	9	3	0	12	0
	AB	0	5	0	0	0	1

Abbreviations: AB, atrial bigeminy; DFA, detrended fluctuation analysis; HR, heart rate; LF, low‐frequency band; HF, high‐frequency band; PAC, premature atrial contractions; pNN50, percentage of successive RR intervals that differ by more than 50 ms; PVC, premature ventricular contractions; RMSSD, root mean square of successive RR interval differences; SDNN, standard deviation of NN intervals.

**FIGURE 3:**
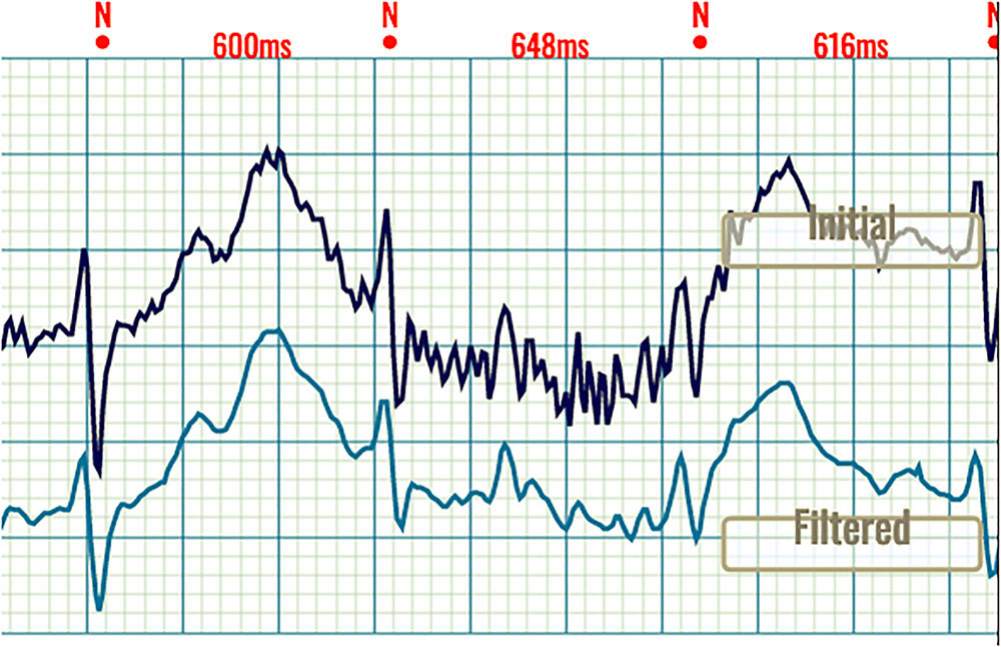
Example of artifacts. Graph was retrieved from the online platform associated with the VITALPATCH device, that is https://csptelemedicinecv.web.app.

**FIGURE 4:**
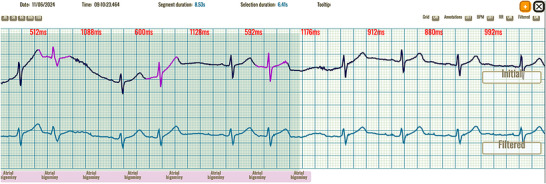
Strip of atrial bigeminies. Graph was retrieved from the online platform associated with the VITALPATCH device, that is https://csptelemedicinecv.web.app.

Atypical episodes included synus bradycardia, synus tachicardia, ventricular ectopies, supraventricular ectopies, supraventricular tachycardia, supraventricular tachyarrhythmia and atrial fibrillation. A lot of synus bradycardia episodes occurred, although those were related to the low resting HR of the subject and were not considered. The other alerts were considered false or highly uncertain, possibly due to electrical noise and movement artifacts (see example in Figure 3), and the physicians suggested verifying the status with a clinical setting. However, the physicians confirmed atrial bigeminies (Figure 4) that appeared only after the expedition on the day between normoxic and hypoxic tests to be true.

**FIGURE 5:**
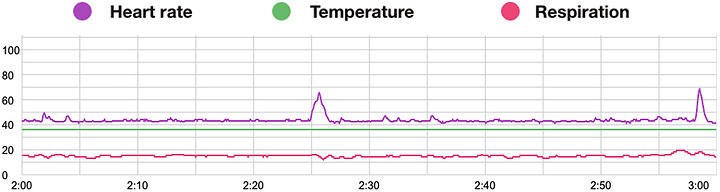
Strip of nocturnal registration with HR peaks. Graph was adapted from the online platform associated with the VITALPATCH device, that is https://csptelemedicinecv.web.app.

During the night, both before and after the expedition, we observed 1–3 events per hour of nocturnal peaks in heart rate (see examples in Figure 5). These episodes may be due to nocturnal movements or apnoeic episodes, and ECG‐reading were furtherly explored by clinicians.

### 3.2 Case series

**FIGURE 6:**
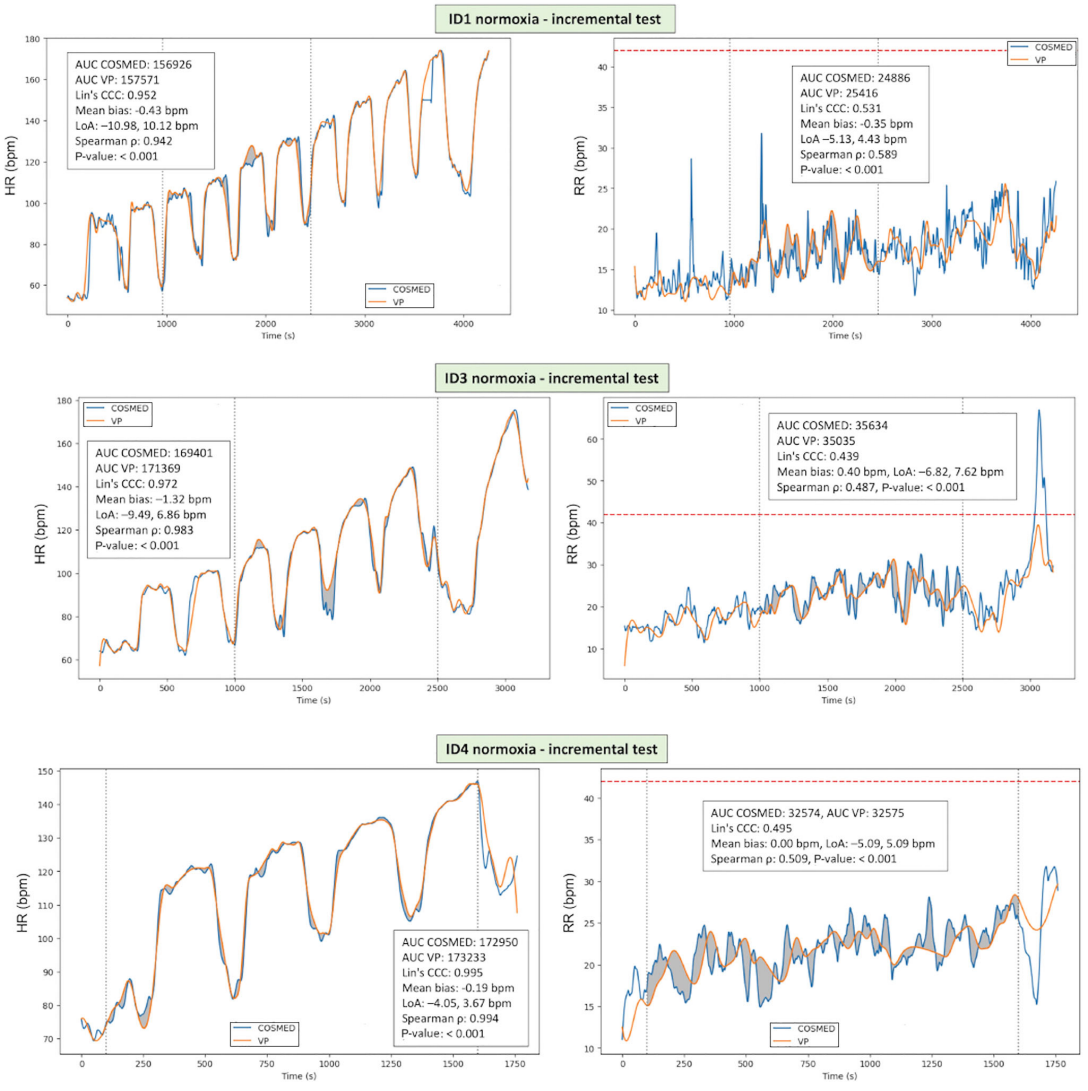
Comparison of heart rate (HR) and respiratory rate (RR) data from VITALPATCH (VP) and COSMED during the incremental test in normoxia. Analytical and statistical values are reported. Vertical lines denote the beginning and the end of the time window considered for the analyses. Horizontal lines in right panels denotes the maximum threshold that the VITALPATCH system applied for RR. Grey area denotes the difference area from the two filtered lines. Calculation and graphs were created with Python.

**FIGURE 7:**
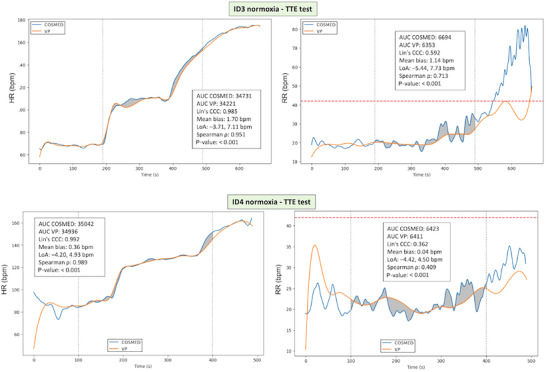
Comparison of heart rate (HR) and respiratory rate (RR) data from VITALPATCH (VP) and COSMED during the time‐to‐exhaustion test in normoxia. Analytical and statistical values are reported. Vertical lines denote the beginning and the end of the time window considered for the analyses. Horizontal lines in right panels denotes the maximum threshold that the VITALPATCH system applied for RR. Grey area denotes the difference area from the two filtered lines. Calculation and graphs were created with Python.

**FIGURE 8:**
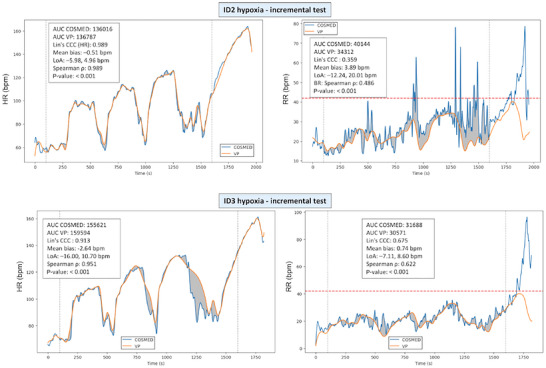
Comparison of heart rate (HR) and respiratory rate (RR) data from VITALPATCH (VP) and COSMED during the incremental test in hypoxia. Analytical and statistical values are reported. Vertical lines denote the beginning and the end of the time window considered for the analyses. Horizontal lines in right panels denotes the maximum threshold that VITALPATCH system applied for RR. Grey area denotes the difference area from the two filtered lines. Calculation and graphs were created with Python.

**FIGURE 9:**
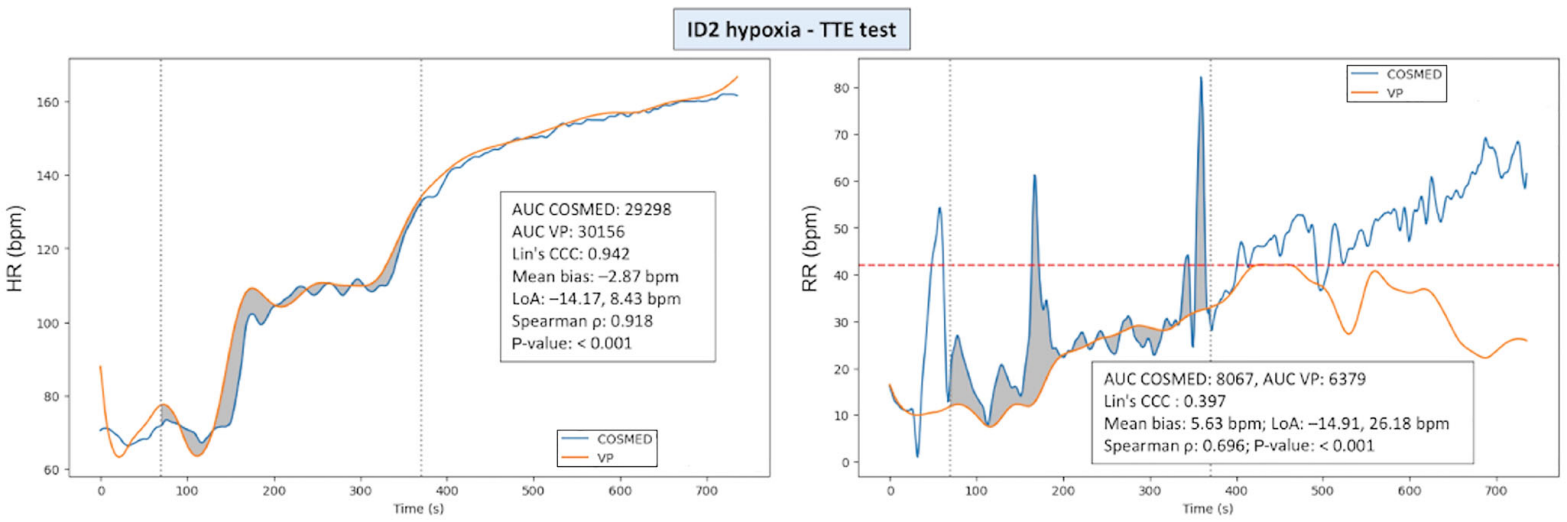
Comparison of heart rate (HR) and respiratory rate (RR) data from VITALPATCH (VP) and COSMED during the time‐to‐exhaustion test in hypoxia. Analytical and statistical values are reported. Vertical lines denote the beginning and the end of the time window considered for the analyses. Horizontal lines in right panels denotes the maximum threshold that VITALPATCH system applied for RR. Grey area denotes the difference area from the two filtered lines. Calculation and graphs were created with Python.

Tables 5 and 6 report the analytical values. The strength of agreement across methods was considered substantial for HR and poor for RR; the AUC of curves filtered from VITALPATCH compared to COSMED remained under 3% of difference, while a large discrepancy emerged concerning RR, with VITALPATCH underestimating values. Overall, greater differences emerged during the TTE test, rather than during the incremental test. From graphs shown in Figures 6, 7, 8, and 9 one can observe the difference areas (highlighted in grey) between COSMED and VITALPATCH filtered data.

**TABLE 5:** Comparison of heart rate data registered from COSMED and VITALPATCH devices.

Heart rate			AUC (%Δ)	CCC	Mean bias (LoA) (bpm)	Spearman's correlation	
Condition	Test	ID				ρ	*P*
Normoxia	Incremental	1	+0.41	0.952	+0.43 (−10.12, +10.98)	0.942	<0.001
		3	+1.16	0.972	+1.32 (−6.86, +9.49)	0.983	<0.001
		4	+0.16	0.995	−0.19 (−3.67, +4.05)	0.994	<0.001
	TTE	3	−1.47	0.985	−1.70 (−7.11, +3.71)	0.951	<0.001
		4	−0.30	0.992	−0.36 (−4.93, +4.20)	0.989	<0.001
Hypoxia	Incremental	2	+0.57	0.989	+0.51 (−4.96, +5.98)	0.989	<0.001
		3	+2.55	0.913	+2.64 (−10.70, +16.00)	0.951	<0.001
	TTE	2	+2.93	0.942	+2.87 (−8.43; +14.17)	0.918	<0.001

Abbreviations: AUC, area under the curve; CCC, Lin's concordance correlation coefficient; LoA, limits of agreement; TTE, time to exhaustion.

**TABLE 6:** Comparison of respiratory rate data registered from COSMED and VITALPATCH devices.

Respiratory rate			AUC (%Δ)	CCC	Mean bias (LoA) (bpm)	Spearman's correlation	
Condition	Test	ID				ρ	*P*
Normoxia	Incremental	1	+2.13	0.531	+0.35 (−4.43; +5.13)	0.589	<0.001
		3	−1.68	0.439	−0.40 (−7.62; +6.82)	0.497	<0.001
		4	0.00	0.495	0.00 (−5.09; +5.09)	0.509	<0.001
	TTE	3	−5.09	0.592	−1.14 (−7.73; +5.44)	0.713	<0.001
		4	−0.19	0.362	−0.04 (−4.50; +4.42)	0.409	<0.001
Hypoxia	Incremental	2	−14.53	0.359	−3.89 (−20.01; +12.24)	0.486	<0.001
		3	−3.52	0.675	−0.74 (−8.60; +7.11)	0.622	<0.001
	TTE	2	−20.92	0.397	−5.63 (−26.18; +14.91)	0.696	<0.001

Abbreviations: AUC, area under the curve; CCC, Lin's concordance correlation coefficient; LoA, limits of agreement; TTE, time to exhaustion.

It should be observed that RR signals obtained from VITALPATCH appear to be considerably smoother than RR signals from COSMED, despite the common filtering algorithm used for both series, thus resulting in poor overall concordance. Therefore, reporting spot values for a given moment from VITALPATCH should not be considered valid. However, the trend of values is almost superimposable, and mean bias was overall acceptable, except for one incremental and one TTE test in the hypoxic condition.

From ECG reports, several alarms were notified (Table 7). However, they were considered false or highly uncertain by the physicians, possibly due to electrical noise and movement artifacts. The report of clinical ECG conducted during the test is shown in Table 8. The physicians highlighted that some events of false paroxysmal supraventricular tachycardia emerged due to artifacts. Altered rhythmic patterns registered during the test for participants ID1 and ID3 (see Table 7) also emerged from 24‐h registration of the VITALPATCH‐registered bipolar ECG. Except for participant ID1, the values of multi‐day ECG and multi‐day HRV reports are plausible.

**TABLE 7:** ECG‐derived metrics from VITALPATCH.

	Normoxia		Hypoxia	
	Incremental	TTE	Incremental	TTE
ID1	1 AF 8 SVTA 73 SVT	No data	No data	No data
	Multi‐day ECG: mean QRS = 11 ms; mean QT_c_ = 51 ms			
	Multi‐day HRV: RMSSD = 142 ms; LF/HF = 0.76			
ID2	No data	No data	4 SVTA 6 VT 6 SVT	1 SVTA 3 VT 13 SVT
	Multi‐day ECG: mean QRS = 81 ms; mean QT_c_ = 311 ms			
	Multi‐day HRV: RMSSD = 111 ms; LF/HF = 2.01			
ID3	18 SVT	3 SVT	1 VB 6 VT 16 SVT	No data
	Multi‐day ECG: mean QRS = 82 ms; mean QT_c_ = 377 ms			
	Multi‐day HRV: RMSSD = 51 ms; LF/HF = 1.5			
ID4	2 SVTA 21 SVT	1 VB 2 SVTA 5 VT 39 SVT	No data	No data
	Multi‐day ECG: mean QRS = 102 ms; mean QT_c_ = 390 ms			
	Multi‐day HRV: RMSSD = 77 ms; LF/HF = 1.55			

Abbreviations: AF, atrial fibrillation; SVT, supraventricular tachycardia; SVTA, supraventricular tachyarrhythmia; TTE, time‐to‐exhaustion; VB, ventricular bigeminy; VT, ventricular tachycardia.

**TABLE 8:** ECG clinic reports during the tests.

	Normoxia		Hypoxia	
	Incremental	TTE	Incremental	TTE
ID1	2 VEBs Data quality: fair	9 VEBs Data quality: low	Pronounced SA 8 VEBs, 1 SVEB Data quality: fair	NA
ID2	NA	NA	No event; Data quality: optimal	NA
ID3	NA	NA	1 VEB Data quality: fair	No event Data quality: fair
ID4	7 SVEBs Data quality: fair	3 SVEBs Data quality: optimal	Lots of artifacts Data quality: low	No event Data quality: fair

Abbreviations: NA, not available; SA, sinus arrhythmia; SVEB, supraventricular ectopic beat; VEB, ventricular ectopic beat.

During the nights, as previously shown with regards to case report's example night strip, on average 1–3 events per hour of nocturnal peaks in heart rate were noticed.

## 4 DISCUSSION

Within the growing field of sensorized patches, the VITALPATCH biosensor previously achieved acceptable accuracies for heart rate, RR and skin temperature in bench testing and in the stationary period (Selvaraj et al., 2018). This study aimed to provide preliminary data on either usefulness or warning for using VITALPATCH in a hypoxic environment.

Compared to a previous validation under hypoxic conditions (Areia et al., 2021), lower agreement was obtained for RR, particularly during the time‐to‐exhaustion test. Our tests were far more stressful than those of the previous validation, and a lot of motion artifacts emerged for the bipolar ECG, which biased the ECG‐related events and the computation of RR. It should be noted that the breath‐by‐breath mode of the COSMED system allows the capture of breath‐by‐breath alterations of the ventilatory pattern, which is inherently not precisely and accurately allowed in the VITALPATCH system.

The possibility of analysing the RR trend (at least up to 42 bpm) still allows the use of VITALPATCH to monitor this vital sign during low‐ and medium‐intensity activity at high altitude. However, this inherent limitation restricts the application of VITALPATCH monitoring during high‐intensity exercise; this limitation becomes even more apparent under hypoxic conditions, where the RR is generally higher in hypoxia compared to normoxia for any given exercise intensity, although this effect exhibits inter‐individual heterogeneity (Bondi & Verratti, 2023; Fornasiero et al., 2019). Heart rate monitoring proves to be valuable, but high body movements lead to motion artifacts on the ECG signal and thus on the reconstruction of HR and in the report of alarms. Considering sleep as a predominantly stationary phase and as highly susceptible to hypoxia, the use of VITALPATCH for sleep monitoring, given the comfort of this biosensor, is of considerable perspective.

The online platform associated with the VITALPATCH device in our experience was useful for real‐data monitoring and data processing. The possibility of obtaining raw data from wearable/portable devices should become a recommendation, since it allows the development of customized analysis and modelling for comparing continuous vital signs from different devices, as we did for this project and released as Supporting information.

### 4.1 Limitations and troubleshooting

Firstly, the design of this study, which involved a limited number of participants and signals, does not permit the provision of definitive conclusions regarding the validity of data acquired during exercise in hypoxia from the VITALPATCH device in comparison to those obtained from the COSMED device. Consequently, a cautious approach should be adopted when interpreting the results. A further limitation pertains to the simulated altitude (approximately 5000 m), which constituted a solitary hypoxic condition and extended well beyond the reported operational range of VITALPATCH.

A limit for the use of VITALPATCH during field studies is the presence of motion artifacts. Artifacts resulted in low data quality, over‐reported events, and possible inaccuracy in electrocardiogram‐derived respiration (EDR). This problem was indeed present in other projects, for example, for computing EDR from a bipolar ECG signal registered from a chest strap (Sbrollini et al., 2022). Artifacts were presumed to be present, given the relative movement of the skin on the underlying tissue, and the presence of clothing that may further create mechanical stimuli on the sensor. The sensor was developed for the purpose of remote monitoring of patients under conditions of near‐absent or otherwise poor motion. It is therefore presumed that its validity in our setting is reduced. It is recommended that future developments in this regard include the implementation of robust filtering algorithms that can either deal with acceptable artifacts or exclude all those time windows in which the recording is unreliable. Another major limit is the maximum value of 42 breaths/min, a limit that as above refers to the use for which the sensor was developed, under conditions where values considerably lower than this threshold are recorded. Moreover, VITALPATCH does not capture $S_{pO_{2}}$, which is of interest during high‐altitude exposure, but it can be coupled to a third sensor.

The comparison of breathing pattern in challenging breathing conditions was biased, as COSMED was set to assess breath‐by‐breath metrics. The smoothing algorithms were set to maintain the fundamental ventilatory pattern, as an excessive smoothing leads to a lack of information that affects analyses aimed at assessing transfer of information and causality relationships from one physiological system to another, the core of network physiology (Bartsch et al., 2015) and which is of interest in hypoxia (Jiang et al., 2021).

The use of recumbent cycling was determined within a broader experimental design, in which multiple physiological measurements were performed simultaneously during exercise. The recumbent cycling modality offers advantages in terms of upper‐body stability and reduced movement artifacts, which is particularly relevant when assessing respiratory parameters with wearable or sensor‐based instruments. This set‐up allowed us to obtain more controlled and reliable measurements under steady conditions. However, different exercise modalities may introduce additional sources of variability, particularly due to increased body movement and changes in breathing patterns. Therefore, future studies should specifically investigate the performance of the instrument across different types of exercise to determine its robustness and validity in more dynamic and ecologically valid conditions.

### 4.2 Perspectives and conclusions

Body patches would be of great usefulness in physiological monitoring at high altitude, as these biosensors reduce challenges of wearable sensors in extreme environments such as extreme cold exposure. In consideration of the potential application of the VITALPATCH sensor in high‐altitude field studies for remote monitoring, the following conclusions can be drawn, although general caution is required given the small size of the sample:

- Hypoxia per se does not affect the quality of the recording.
- The integrity of the recording for reporting ECG events is significantly compromised by the presence of artefacts, which usually lead to over‐reporting; the implementation of novel preprocessing filtering algorithms has the potential to enhance the reliability of the data.
- The process of heart rate monitoring is superimposable on that of a chest strap.
- Spot values, as well as minimum, maximum and mean values, from RR monitoring are unreliable in circumstances necessitating modified breathing patterns (e.g., maximal exertion where breath is occasionally held) or conditions where RRs approaching 42 bpm are attained; the employment of novel algorithms for EDR, incorporating conservative filtering to eliminate unreliable windows, has the potential to enhance data reliability.
- In resting conditions and in those efforts which are submaximal and not greatly pulsatile, both heart rate and respiration rate monitoring have been shown to be reliable and informative; this lends weight to the use for monitoring sleep.
- Although the features of VITALPATCH do not allow the capture of breath‐by‐breath ventilatory patterns, the overall trend can allow the use of RR for network physiology approaches.
- Given the reliability criteria are met, the possibility of automatically obtaining 24‐h ECG data of clinically relevant intervals (such as QT) and a HRV comprehensive report are useful features.
- Temperature and motion monitoring, which were not the focus of the present study, remain a useful opportunity for expeditions in extreme environments.
- The device in its current form is not suitable for mountain rescue.

The following section outlines a potential workflow to optimize the use of VITALPATCH during high altitude studies:

- ensuring that recent stress ECGs are obtained for each subject, accompanied by a clinical report;
- structuring of a pre‐test, conducted by using validated tools for the monitoring of cardiac and respiratory response during rest and incremental testing, so as to create reliable references for comparison;
- prior to application of the sensor, skin must be meticulously cleansed and shaved to ensure optimal adhesion, and the utilization of the additional cover is recommended to enhance the adhesion of the patch to the chest;
- regular monitoring of the data from the online platform in order to ascertain the plausibility of the recordings and to ascertain the battery levels, which permit approximately 7 days of recording;
- being mindful of the fact that the platform is prone to reporting erroneous data when RRs exceed 42 beats/min;
- during movement, relying on data trend for RR, rather than on spot values
- consulting a cardiology specialist in order to verify the functionality of the alarms;
- creating 24‐h or multi‐day reports for ECG and HRV;
- downloading the raw data of a selected time period, an option which is always available from the associated platform, with a view to developing independent filtering and analyses.

## AUTHOR CONTRIBUTIONS

Conceptualization: Danilo Bondi and Anna Pedrinolla. Methodology: Danilo Bondi and Anna Pedrinolla. Software: Danilo Bondi; Formal Analysis: Danilo Bondi and Anna Pedrinolla. Investigation: Ferdinando Calcagno, Elisa Fioraso, and Anna Pedrinolla. Validation: Danilo Bondi. Writing—Original Draft: Danilo Bondi and Anna Pedrinolla. Writing—Review & Editing: Ferdinando Calcagno, Elisa Fioraso, Camillo Di Giulio, and Federico Schena. Funding Acquisition: Anna Pedrinolla. Resources: Danilo Bondi, Camillo Di Giulio, Federico Schena, and Anna Pedrinolla. Data curation: Danilo Bondi and Anna Pedrinolla. Visualization: Danilo Bondi. Supervision: Camillo Di Giulio. Project Administration: Federico Schena and Anna Pedrinolla. All authors have read and approved the final version of this manuscript and agree to be accountable for all aspects of the work in ensuring that questions related to the accuracy or integrity of any part of the work are appropriately investigated and resolved. All persons designated as authors qualify for authorship, and all those who qualify for authorship are listed.

## CONFLICT OF INTEREST

None declared.

## FUNDING INFORMATION

None.

## Supporting information



Phyton code for signals comparison.

## Data Availability

Data will be available upon reasonable request.
