# Supplementary material for: Advancing tele‐physiology: A chest patch solution for continuous, non‐invasive remote monitoring in a hypoxic environment
Source: Exp Physiol. 2026 May 30;111(7):3280–93. doi: 10.1113/EP093718 (PMC13327313; doi:10.1113/EP093718)
Supplement: Supplementary file 1 — Phyton code for signals comparison. [file EPH-111-3280-s001.rtf]

# PREPARATION: install libraries if necessaryimport pandas as pdimport numpy as npimport matplotlib.pyplot as pltimport statsmodels.api as smfrom scipy.interpolate import interp1dfrom scipy.signal import savgol_filterfrom sklearn.metrics import aucfrom scipy.stats import linregressfrom scipy.stats import spearmanrdurata_del_test_COSMED_minuti = … # insert duration (minutes)durata_del_test_COSMED_secondi = … # insert duration (seconds)durata_del_test_COSMED = durata_del_test_COSMED_minuti * 60 + durata_del_test_COSMED_secondidfVP = pd.read_excel(‘….xlsx', sheet_name=‘sheet’_name) # insert Excel file name and Excel sheet namedfVP['Time'] = pd.to_datetime(dfVP['Time'], unit='ms') dfVP['Tempo'] = (dfVP['Time'] - dfVP['Time'][0]).dt.total_seconds()dfCOSMED = pd.read_excel(‘….xlsx', sheet_name='Dati', header=0, skiprows=range(1,3)) # insert Excel file name and Excel sheet namedfCOSMED['Tempo'] = pd.to_datetime(dfCOSMED['t'], format='%H:%M:%S')dfCOSMED['Tempo'] = (dfCOSMED['Tempo'] - dfCOSMED['Tempo'].iloc[0]).dt.total_seconds().astype(int)dfVP = dfVP.sort_values(by=['Tempo'])dfVP = dfVP.drop_duplicates(subset=['Tempo'])# adjust manually start and end of the signalinizio_test_COSMED = 0 # (seconds), adjust if neededfine_test_COSMED = durata_del_test_COSMEDtaglio_VP = … # s, insert end of time window to be cutinizio_test_VP = inizio_test_COSMED + taglio_VPfine_test_VP = fine_test_COSMED + taglio_VPdfVP = dfVP[(dfVP['Tempo'] >= inizio_test_VP) & (dfVP['Tempo'] <= fine_test_VP)]dfCOSMED = dfCOSMED[(dfCOSMED['Tempo'] >= inizio_test_COSMED) & (dfCOSMED['Tempo'] <= fine_test_COSMED)]dfVP.loc[:, 'Tempo'] = dfVP['Tempo'] - dfVP['Tempo'].min()dfCOSMED.loc[:, 'Tempo'] = dfCOSMED['Tempo'] - dfCOSMED['Tempo'].min()# imputation for VitalPatch dataidxBR = dfVP['BR'].notna()idxHR = dfVP['HR'].notna()lowessBR = sm.nonparametric.lowess(dfVP['BR'][idxBR], dfVP['Tempo'][idxBR], frac=0.001) # Regulate frac if neededlowessHR = sm.nonparametric.lowess(dfVP['HR'][idxHR], dfVP['Tempo'][idxHR], frac=0.001) # Regulate frac if neededfBR = interp1d(lowessBR[:, 0], lowessBR[:, 1], kind='cubic', fill_value='extrapolate')fHR = interp1d(lowessHR[:, 0], lowessHR[:, 1], kind='cubic', fill_value='extrapolate')dfVP['BR_imputed'] = dfVP['BR'].copy()dfVP.loc[~idxBR, 'BR_imputed'] = fBR(dfVP.loc[~idxBR, 'Tempo'])dfVP['HR_imputed'] = dfVP['HR'].copy()dfVP.loc[~idxHR, 'HR_imputed'] = fHR(dfVP.loc[~idxHR, 'Tempo'])# Smoothing (Savitzky-Golay filter)window_length_COSMED = 10  # adjust window length as neededwindow_length_VP = window_length_COSMED * 15 # sampling interval between rows in VP are lower than COSMEDpolyorder = 3  # Adjust polynomial order as neededdfCOSMED['Rf_smooth'] = savgol_filter(dfCOSMED['Rf'], window_length_COSMED, polyorder)dfCOSMED['HR_smooth'] = savgol_filter(dfCOSMED['HR'], window_length_COSMED, polyorder)dfVP['HR_imputed_smooth'] = savgol_filter(dfVP['HR_imputed'], window_length_VP, polyorder)dfVP['BR_imputed_smooth'] = savgol_filter(dfVP['BR_imputed'], window_length_VP, polyorder)# graphsfig, axes = plt.subplots(2, 1, figsize=(10, 12), sharex=True)# HR Comparisonaxes[0].plot(dfCOSMED['Tempo'], dfCOSMED['HR_smooth'], label='COSMED HR')axes[0].plot(dfVP['Tempo'], dfVP['HR_imputed_smooth'], label='VP HR')axes[0].set_ylabel('bpm')axes[0].set_ylim(40, 200)axes[0].set_title('HR Comparison')axes[0].legend()axes[0].grid(False)# BR Comparisonaxes[1].plot(dfCOSMED['Tempo'], dfCOSMED['Rf_smooth'], label='COSMED BR')axes[1].plot(dfVP['Tempo'], dfVP['BR_imputed_smooth'], label='VP BR')axes[1].set_xlabel('Time (s)')axes[1].set_ylabel('bpm')axes[1].set_ylim(8, 42)axes[1].set_title('BR Comparison')axes[1].legend()axes[1].grid(False)plt.tight_layout()plt.show()# resamplingt_start = dfCOSMED['Tempo'].min()t_end = dfCOSMED['Tempo'].max()resampling_interval = 1  # seconds, adjustnew_time = np.arange(t_start, t_end, resampling_interval)dfCOSMED_resampled = pd.DataFrame({'Tempo': new_time})resBR_C = interp1d(dfCOSMED['Tempo'], dfCOSMED['Rf_smooth'], kind='linear', fill_value='extrapolate')resHR_C = interp1d(dfCOSMED['Tempo'], dfCOSMED['HR_smooth'], kind='linear', fill_value='extrapolate')dfCOSMED_resampled['BR_res'] = resBR_C(dfCOSMED_resampled['Tempo'])dfCOSMED_resampled['HR_res'] = resHR_C(dfCOSMED_resampled['Tempo'])t_start_vp = dfVP['Tempo'].min()t_end_vp = dfVP['Tempo'].max()new_time_vp = np.arange(t_start_vp, t_end_vp, 1)  # 1 sdfVP_resampled = pd.DataFrame({'Tempo': new_time_vp})resBR_VP = interp1d(dfVP['Tempo'], dfVP['BR_imputed_smooth'], kind='linear', fill_value='extrapolate')resHR_VP = interp1d(dfVP['Tempo'], dfVP['HR_imputed_smooth'], kind='linear', fill_value='extrapolate')dfVP_resampled['BR_res'] = resBR_VP(dfVP_resampled['Tempo'])dfVP_resampled['HR_res'] = resHR_VP(dfVP_resampled['Tempo'])window_length_gen = 10  # Adjust window length as neededpolyorder_gen = 3  # Adjust polynomial order as neededdfCOSMED_resampled['BR_res'] = savgol_filter(dfCOSMED_resampled['BR_res'], window_length_gen, polyorder_gen)dfCOSMED_resampled['HR_res'] = savgol_filter(dfCOSMED_resampled['HR_res'], window_length_gen, polyorder_gen)dfVP_resampled['HR_res'] = savgol_filter(dfVP_resampled['HR_res'], window_length_gen, polyorder_gen)dfVP_resampled['BR_res'] = savgol_filter(dfVP_resampled['BR_res'], window_length_gen, polyorder_gen)taglio_inizio = … # (seconds) select start of time window for calculationstaglio_fine = … # (seconds) select end of time window for calculationsdfCOSMED_res_sliced = dfCOSMED_resampled[taglio_inizio:taglio_fine]dfVP_res_sliced = dfVP_resampled[taglio_inizio:taglio_fine]#AUCaucCOSMED_BR = np.trapezoid(dfCOSMED_res_sliced['BR_res'], x=dfCOSMED_res_sliced['Tempo'])aucCOSMED_HR = np.trapezoid(dfCOSMED_res_sliced['HR_res'], x=dfCOSMED_res_sliced['Tempo'])aucVP_BR = np.trapezoid(dfVP_res_sliced['BR_res'], dfVP_res_sliced['Tempo'])aucVP_HR = np.trapezoid(dfVP_res_sliced['HR_res'], dfVP_res_sliced['Tempo'])print(f"AUC COSMED BR: {aucCOSMED_BR}")print(f"AUC VP BR: {aucVP_BR}")print(f"AUC COSMED HR: {aucCOSMED_HR}")print(f"AUC VP HR: {aucVP_HR}")#CCC with Lin’s methoddef concordance_correlation_coefficient(y_true, y_pred):    mean_true = np.mean(y_true)    mean_pred = np.mean(y_pred)    sd_true = np.std(y_true)    sd_pred = np.std(y_pred)    covariance = np.cov(y_true, y_pred, ddof=0)[0, 1]    numerator = 2 * covariance    denominator = sd_true**2 + sd_pred**2 + (mean_true - mean_pred)**2    ccc = numerator / denominator    return cccccc_br = concordance_correlation_coefficient(dfCOSMED_res_sliced['BR_res'], dfVP_res_sliced['BR_res'])print(f"Lin's CCC (BR): {ccc_br}")ccc_hr = concordance_correlation_coefficient(dfCOSMED_res_sliced['HR_res'], dfVP_res_sliced['HR_res'])print(f"Lin's CCC (HR): {ccc_hr}")# mean biasdifferencesBR = dfCOSMED_res_sliced['BR_res'] - dfVP_res_sliced['BR_res']meansBR = (dfCOSMED_res_sliced['BR_res'] + dfVP_res_sliced) / 2mean_biasBR = np.mean(differencesBR)std_diffBR = np.std(differencesBR, ddof=1)loa_upperBR = mean_biasBR + 1.96 * std_diffBRloa_lowerBR = mean_biasBR - 1.96 * std_diffBRprint(f"mean bias BR: {mean_biasBR}")print(f"loa upper BR: {loa_upperBR}")print(f"loa lower BR: {loa_lowerBR}")differencesHR = dfCOSMED_res_sliced['HR_res'] - dfVP_res_sliced['HR_res']meansHR = (dfCOSMED_res_sliced['HR_res'] + dfVP_res_sliced) / 2mean_biasHR = np.mean(differencesHR)std_diffHR = np.std(differencesHR, ddof=1)loa_upperHR = mean_biasHR + 1.96 * std_diffHRloa_lowerHR = mean_biasHR - 1.96 * std_diffHRprint(f"mean bias HR: {mean_biasHR}")print(f"loa upper HR: {loa_upperHR}")print(f"loa lower HR: {loa_lowerHR}")# Spearman correlationfrom scipy.stats import spearmanrrho, p_value = spearmanr(dfCOSMED_res_sliced['HR_res'], dfVP_res_sliced['HR_res'])print(f"HR: Spearman ρ {rho:.3f}, p-value: {p_value:.5f}")rho, p_value = spearmanr(dfCOSMED_res_sliced['BR_res'], dfVP_res_sliced['BR_res'])print(f"BR: Spearman ρ {rho:.3f}, p-value: {p_value:.5f}")# final graphsplt.figure(figsize=(10, 6))plt.plot(dfCOSMED_resampled['Tempo'], dfCOSMED_resampled['HR_res'], label='COSMED HR (Resampled)')plt.plot(dfVP_resampled['Tempo'], dfVP_resampled['HR_res'], label='VP HR (Resampled)')plt.xlabel('Time (s)')plt.ylabel('bpm')plt.title('filtered HR')plt.axvline(x=taglio_inizio, color='grey', linestyle='dotted')plt.axvline(x=taglio_fine, color='grey', linestyle='dotted')plt.fill_between(dfCOSMED_resampled['Tempo'], dfCOSMED_resampled['HR_res'], dfVP_resampled['HR_res'],                 where=(dfCOSMED_resampled['Tempo'] >= taglio_inizio) & (dfCOSMED_resampled['Tempo'] <= taglio_fine),                 color='grey', alpha=0.5)plt.legend()plt.grid(False)plt.show()plt.figure(figsize=(10, 6))plt.plot(dfCOSMED_resampled['Tempo'], dfCOSMED_resampled['BR_res'], label='COSMED BR (Resampled)')plt.plot(dfVP_resampled['Tempo'], dfVP_resampled['BR_res'], label='VP BR (Resampled)')plt.axhline(y=42, color='r', linestyle='--') # Added line for y=42plt.xlabel('Time (s)')plt.ylabel('bpm')plt.title('filtered BR')plt.axvline(x=taglio_inizio, color='grey', linestyle='dotted')plt.axvline(x=taglio_fine, color='grey', linestyle='dotted')plt.fill_between(dfCOSMED_resampled['Tempo'], dfCOSMED_resampled['BR_res'], dfVP_resampled['BR_res'],                 where=(dfCOSMED_resampled['Tempo'] >= taglio_inizio) & (dfCOSMED_resampled['Tempo'] <= taglio_fine),                 color='grey', alpha=0.5)plt.legend()plt.grid(False)plt.show()
